# Assessing self-administration of medication: video-based evaluation of patient performance in the ABLYMED study

**DOI:** 10.3389/fmed.2024.1444567

**Published:** 2024-11-19

**Authors:** Anneke Luegering, Robert Langner, Stefan Wilm, Thorsten R. Doeppner, Dirk M. Hermann, Helmut Frohnhofen, Janine Gronewold

**Affiliations:** ^1^Hospital Pharmacy, University Hospital Düsseldorf, Düsseldorf, Germany; ^2^Institute of Systems Neuroscience, University Hospital Düsseldorf, Düsseldorf, Germany; ^3^Institute of Neuroscience and Medicine (INM-7: Brain and Behaviour), Research Centre Jülich, Jülich, Germany; ^4^Institute of General Practice, Centre for Health and Society (chs), University Hospital Düsseldorf, Düsseldorf, Germany; ^5^Department of Neurology, University Hospital Giessen, Giessen, Germany; ^6^Research Institute for Health Sciences and Technologies (SABITA), Istanbul Medipol University, Istanbul, Türkiye; ^7^Department of Anatomy and Cell Biology, Medical University of Varna, Varna, Bulgaria; ^8^Department of Neurology, University of Göttingen, Göttingen, Germany; ^9^Department of Neurology and Center for Translational Neuro- and Behavioral Sciences (C-TNBS) University Hospital Essen, University Duisburg-Essen, Essen, Germany; ^10^Department of Orthopedics and Trauma Surgery, University Hospital Düsseldorf, Düsseldorf, Germany; ^11^Department of Medicine, Geriatrics, Faculty of Health, Witten, Germany

**Keywords:** self-administration, aged, medication management problems, video recordings, administration steps

## Abstract

**Background:**

Older adults often face challenges in medication management due to multimorbidity and complex medication regimens, which frequently go unreported. Unrecognized problems, however, may lead to a loss of drug efficacy and harmful side effects. This study aimed to quantify the prevalence of such problems by applying a novel video-based assessment procedure in a sample of elderly patients.

**Methods:**

In this study, 67 elderly in-patients (≥70 years old and regularly taking ≥5 different drugs autonomously) from the ABLYMED study participated in a placebo-based assessment of medication management with five different dosage forms in an instructed manner while being filmed. Patient performance was quantified by the median value of two raters who evaluated each step of medication administration, which were summed to sum scores for each dosage form and an overall impression for each dosage form with a standardized and previously validated rating scheme.

**Results:**

The median (Q1;Q3) sum score for tablets was 7.0 (5.0;8.0) with a theoretical range between 4.0 and 17.0, for eye-drops 2.0 (1.0;2.0) with a theoretical range between 1.0 and 5.0, for oral drops 4.0 (3.0;6.0) with a theoretical range between 3.0 and 12.0, for pens 7.0 (5.0;9.0) with a theoretical range between 4.0 and 17.0 and for patches 5.0 (4.0;7.0) with a theoretical range between 3.0 and 15.0. The most difficult step of medication administration was peeling off the protective liner of a patch: 30% had severe difficulties or it was not possible, 21% had moderate difficulties and 49% had mild or no difficulties.

**Discussion:**

In a sample of patients with autonomous medication management, our novel assessment procedure identified a substantial fraction of patients with handling problems for each dosage form. This suggests that patients´ medication management problems should be assessed regularly in clinical routine and tackled by patient-individual training or modification of the prescribed drug regimens to achieve effective drug therapy in the elderly.

## Introduction

Older patients often receive multiple drugs simultaneously as a consequence of the accumulation of age-related diseases, such as hypertension, diabetes, and heart failure, which require combinations of drugs in different dosage forms like tablets and pens (1–3). These complex medication regimens impose considerable challenges to many patients (4). Moreover, multimorbidity and aging are associated with functional and cognitive impairments, making medication self-management even more challenging. Thus, medication self-management can be inadequate, and unintentional non-adherence to pharmacological treatment regimens can result from these challenges. Such non-adherence is specifically a problem in independently living patients receiving multiple drugs and often remains unrecognized by responsible health care practitioners (5–7). In fact, the patients’ skills to manage drug regimens are frequently overrated by practitioners (8).

To prevent unintentional non-adherence and associated harms, health care providers should evaluate their patients’ medication self-management skills (9). Compromised ability to manage medications should lead to the modification of prescribed drug regimens, unless specific trainings can improve the patients’ drug handling skills or other types of support (e.g., by family members or nurses) is provided (10). There are several ways to assess the patients’ medication self-management skills. The simplest one is to ask patients about their experience with medication handling. Such interviews require only a short amount of time and no equipment but may lead to invalid assessments if patients provide biased answers. Reasons for invalid patient self-report can be a lack of knowledge about the medication, memory problems, overconfidence, social desirability and resilience. In contrast, observing a patient’s medication management performance, rather than relying on self-report, may considerably increase the validity (8) of the assessment. Ideally, judgment about medication management skills should not depend on one person (11).

To the best of our knowledge, no previous study has assessed elderly patients’ medication management performance in a comprehensive and realistic test scenario (12) using such an observational approach. Previous studies were limited to the examination of specific dosage forms, for instance only assessing eye-drop instillation (13). Another limitation of previous studies is that medication management skills were evaluated by one person only. For example, the instrument MedMaIDE (Medication Management Instrument for Deficiencies in the Elderly) examines the observation by one person and assesses domains such as filling a glass with water, removing the top from a medication container, counting out a required number of pills into the hand or a cup, administering the medication and sipping enough water to swallow the medication (14). Another study describing a video-based performance measure of medication management from Schenk et al. evaluated problems in medication management only qualitatively (8).

The main object of the “ability to self-administer medication in non-demented in-hospital patients” (ABLYMED) study is the development of a novel tool to assess the ability to self-administer medication in hospitalized patients (15). The assessment contains self-report measures and a video-based performance measure (11, 16).

Here, we describe the results of the video-based performance measure of the self-administration of medication in different dosage forms, which are one part of the ABLYMED study. We aimed to quantify the prevalence of medication management problems by applying a novel video-based assessment procedure in a sample of elderly patients.

For a comprehensive analysis of the video-based performance measure, we evaluated all common dosage forms using placebo medications in their original packaging. Patients were filmed while performing medication handling in an instructed manner. The videos were then evaluated by multiple raters at various time points to achieve an objective, reliable, and valid evaluation of the patients’ ability to self-administer medication (11, 15).

## Materials and methods

### Setting

The ABLYMED cross-sectional single-center observational study included 100 non-demented patients from the Department of Orthopedics and Trauma Surgery and the Department of Vascular and Endovascular Surgery of the University Hospital Duesseldorf ≥70 years of age regularly taking ≥5 different drugs autonomously (09/2021–04/2022) (15). During the continuous recruitment, all eligible patients received an information brochure and were asked to participate in the study by the principal investigator after arrival at the hospital. After informed consent was given and signed by the patient, he/she entered the study. The median age was 79 years (Q1;Q3 = 74;84 years) and 50% were female. Patients’ performance of medication management was examined using placebo drugs. For this, patients were filmed with a smart phone camera while managing medication in five different dosage forms (tablets, eye-drops, oral drops, pens and patches) in an instructed way in the patient room. The video recordings were made by one person (AL, pharmacist) to reduce stress for the patients. The medication management performance task took on average about 20 min. The video recorded performance was rated by two independent raters via a systematic and previously validated evaluation procedure (11, 15). Of the 100 patients included into the ABLYMED study, 67 agreed to the video recordings and 57 patients completed the management of all dosage forms. Reasons for non-participation in the video-recorded performance tasks included a lack of motivation to spend the time required, feeling too sick, not wanting to be filmed, and getting a visit. Besides, creating an undisturbed atmosphere in the patient room was sometimes not possible. The study was approved by the ethics committee at the medical faculty of the Heinrich Heine University Duesseldorf (reference number 2021–1435). Before participating in the study, all patients gave written informed consent.

### Video evaluation

Five videos were recorded of each patient (one per dosage form: tablets, eye-drops, oral drops, pen and patches). The video recordings were done by AL (pharmacist) and independently evaluated by JG (psychologist and epidemiologist) and TD (neurologist) using a standardized assessment form, rating rules and a training developed by experts in geriatrics. The training included explanations to the instructional videos, which were used to instruct the patients, to the standardized assessment form, and to the rating rules. Furthermore, any questions of the raters could be clarified.

The assessment form consisted of 5-point Likert-type rating scales for each step of the medication administration (5 = not possible, i.e., practical assistance needed or interruption; 4 = severe difficulties, i.e., execution hardly possible or success of therapy at risk; 3 = moderate difficulties, i.e., execution significantly slowed down; 2 = mild difficulties, i.e., execution slightly slowed down; 1 = no difficulties, i.e., correct and fluid execution). Besides, in some cases, the assessment form allowed the selection between correct or incorrect (1 = correct and 2 = incorrect). Patients were defined as suffering from handling problems if the step of medication administration was rated with severe difficulties, not possible or incorrect administration. (see Table 1).

**Table 1:** Rating results of each step of medication administration.

Dosage form	N (%)	Median (Q1;Q3)
Tablets		
White tablet removal from the blister pack	No difficulties (1): 19 (29.2%) Mild difficulties (2): 28 (43.1%) Moderate difficulties (3): 14 (21.5%) Severe difficulties (4): 4 (6.2%) Not possible (5): 0 (0.0%)	2.0 (1.0;3.0)
Blue tablet removal from the tablet tube	No difficulties (1): 20 (30.8%) Mild difficulties (2): 23 (35.4%) Moderate difficulties (3): 16 (24.6%) Severe difficulties (4): 3 (4.6%) Not possible (5): 3 (4.6%)	2.0 (1.0;3.0)
Cutting the blue tablet	No difficulties (1): 27 (41.5%) Mild difficulties (2): 13 (20.0%) Moderate difficulties (3): 12 (18.5%) Severe difficulties (4): 8 (12.3%) Not possible (5): 5 (7.7%)	2.0 (1.0;3.0)
Correctly filling the pill organizer	Yes (1): 45 (69.2%) No (2): 20 (30.8%)	1.0 (1.0;2.0)
Eye-drops		
Open the one-dose ophtiole dispenser	No difficulties (1): 31 (48.4%) Mild difficulties (2): 24 (37.5%) Moderate difficulties (3): 5 (7.8%) Severe difficulties (4): 3 (4.7%) Not possible (5): 1 (1.6%)	2.0 (1.0;2.0)
Oral drops		
Open the child-resistant dropper bottle	No difficulties (1): 39 (60.0%) Mild difficulties (2): 12 (18.5%) Moderate difficulties (3): 4 (6.2%) Severe difficulties (4): 3 (4.6%) Not possible (5): 7 (10.8%)	1.0 (1.0;2.0)
Aiming at the teaspoon	No difficulties (1): 41 (63.1%) Mild difficulties (2): 9 (13.8%) Moderate difficulties (3): 11 (16.9%) Severe difficulties (4): 2 (3.1%) Not possible (5): 2 (3.1%)	1.0 (1.0;2.0)
Correct number of drops (n = 10) on the teaspoon	Yes (1): 41 (63.1%) No (2): 24 (36.9%)	1.0 (1.0;2.0)
Pen		
Remove the transparent cap of the pen	No difficulties (1): 54 (88.5%) Mild difficulties (2): 3 (4.9%) Moderate difficulties (3): 3 (4.9%) Severe difficulties (4): 1 (1.6%) Not possible (5): 0 (0.0%)	1.0 (1.0;1.0)
Remove the green cap of the needle	No difficulties (1): 31 (50.8%) Mild difficulties (2): 8 (13.1%) Moderate difficulties (3): 18 (29.5%) Severe difficulties (4): 1 (1.6%) Not possible (5): 3 (4.9%)	1.0 (1.0;3.0)
Dialing in the right dose (12 units)	No difficulties (1): 24 (39.3%) Mild difficulties (2): 11 (18.0%) Moderate difficulties (3):11 (18.0%) Severe difficulties (4): 13 (21.3%) Not possible (5): 2 (3.3%)	2.0 (1.0;3.5)
Injection into a ball	No difficulties (1): 32 (52.5%) Mild difficulties (2): 12 (19.7%) Moderate difficulties (3): 8 (13.1%) Severe difficulties (4): 4 (6.6%) Not possible (5): 5 (8.2%)	1.0 (1.0;3.0)
Patches		
Unpacking the patch	No difficulties (1): 38 (60.3%) Mild difficulties (2): 17 (27.0%) Moderate difficulties (3): 5 (7.9%) Severe difficulties (4): 1 (1.6%) Not possible (5): 2 (3.2%)	1.0 (1.0;2.0)
Peeling off the protective liner	No difficulties (1): 11 (17.5%) Mild difficulties (2): 20 (31.7%) Moderate difficulties (3): 13 (20.6%) Severe difficulties (4): 11 (17.5%) Not possible (5): 8 (12.7%)	3.0 (2.0;4.0)
Applying the patch onto the skin	No difficulties (1): 38 (60.3%) Mild difficulties (2): 14 (22.2%) Moderate difficulties (3): 5 (7.9%) Severe difficulties (4): 0 (0.0%) Not possible (5): 6 (9.5%)	1.0 (1.0;2.0)

Data are shown as number (%), median (Q1;Q3).

In addition, the assessment form contained a rating of the overall impression of the medication management performance observed for each dosage form (5 = very poor: inadequate handling and incorrect result; 4 = poor: inadequate handling or incorrect result; 3 = fair: support needed to overcome difficulties; 2 = good: no support needed despite minor difficulties; 1 = very good: no support needed and fluid execution; see Table 2). Patients were defined as suffering from handling problems if the overall impression was four or five.

**Table 2:** Sum scores of each dosage form.

Dosage form	Median (Q1;Q3)	Range	Theoretical range	N
Tablets	7.0 (5.0;8.0)	4.0–15.0	4.0–17.0	65
Eye-drops	2.0 (1.0;2.0)	1.0–5.0	1.0–5.0	64
Oral drops	4.0 (3.0;6.0)	3.0–12.0	3.0–12.0	65
Pen	7.0 (5.0;9,5)	4.0–17.0	4.0–20.0	61
Patches	5.0 (4.0;7.0)	3.0–15.0	3.0–15.0	63

Data are shown as median (Q1;Q3), (theoretical) score range and number of participants included in the analysis per dosage form.

### Statistical analysis

The evaluation procedure of the video recordings has been previously shown to be valid and reproducible (11). The dosage form-specific interrater agreement between the two raters (concerning the continuous sum score for each dosage form resulting from summing up the scores for each administration step for each dosage form for each rater) was satisfactory with an intraclass correlation coefficient (ICC) between 0.67 and 0.99. The agreement between the reference standard and the two raters was satisfactory as well with an ICC between 0.52 and 1.00. Further, the intra-rater agreement over time (retest reliability) for the two raters was excellent with an ICC of 1.00 for JG and an ICC of 0.97 for TD (11). The interrater agreement for the ratings of each step of medication administration and the overall impression of each dosage form is shown as weighted Cohen’s kappa (linear weighting) and as the differences between rating results from rater JG and rater TD (see Supplementary Tables S1, S2). For both the evaluation of each step of the medication administration and the overall impression, the median value of the two raters was calculated and rounded up to the next whole number to summarize the ratings of the two raters. That means if there was a discrepancy of one between the raters, the most severe rating was taken. Besides, the median value rounded up to the next whole number was calculated for the summed-up scores for each administration step for each dosage form for each rater. Thus, we achieved five sum scores per patient, one for each dosage form, which quantified patients’ performance for each dosage form. Descriptive results are reported as median (Q1;Q3).

To evaluate the correlation between the sum score of each dosage form and the overall impression, Kendall’s-Tau correlation analyses were performed. Data were analyzed using SPSS 22 for Windows (IBM Corporation, Armonk, NY, United States).

## Results

Of the 100 patients included into the ABLYMED study, 67 agreed to the video recordings and 57 patients completed the management of all dosage forms. The median age of the patients who agreed to the video recordings was 78 years (Q1;Q3 = 73;83 years) and 49.2% were female.

The rating results of each step of the medication administration are presented in Table 1. For tablets, the median score for each administration step was between 1.0 and 2.0, meaning no or mild difficulties. The administration step of cutting the tablets [median 2.0 (1.0;3.0)] was the most difficult: 20% of the patients had severe difficulties with cutting the tablets or did not manage it at all (score 4 or 5). For eye-drops, the median score for opening the dispenser was 2.0 (1.0;2.0): most patients (86%) had no or only mild difficulties. For oral drops, the median score for each administration step was 1.0; still, 16% of the patients had severe difficulties or were not able to open the child-resistant dropper bottle. For pens, the median score for each administration step was between 1.0 and 2.0. The most difficult administration step was dialing in the right dose [median 2.0 (1.0;3.5)]: 24% of the patients had severe difficulties with dialing in the right dose or did not manage it at all. For patches, the median score for each administration step was between 1.0 and 3.0. The most difficult step was peeling off the protective liner [median 3.0 (2.0;4.0)]: 30% of the patients had severe problems with removing the protective liner or were completely unable to peel it off. The percentage of patients with handling problems in at least one step of administration (severe difficulties, not possible or incorrect administration) was 46% for tablets, 6% for eye-drops, 42% for oral drops, 31% for pens and 30% for patches. 84% of patients had handling problems in at least one step of administration for at least one dosage form.

Table 2 shows the sum scores of all administration steps for each dosage form. The median sum score quantifies the performance of each patient per dosage form. The median sum score for tablets was 7.0 (5.0;8.0) with a theoretical range between 4.0 and 17.0, for eye-drops 2.0 (1.0;2.0) with a theoretical range between 1.0 and 5.0, for oral drops 4.0 (3.0;6.0) with a theoretical range between 3.0 and 12.0, for pens 7.0 (5.0;9,5) with a theoretical range between 4.0 and 17.0 and for patches 5.0 (4.0;7.0) with a theoretical range between 3.0 and 15.0.

The overall impression ratings for each dosage form are presented in Table 3. Tablets [median 3.0 (2.0;4.0)] and pens [median 3.0 (2.0;4.0)] achieved the worst overall impression ratings, followed by patches [median 2.0 (2.0;4.0)], oral drops [median 2.0 (1.0;3.0)] and eye-drops [median 2.0 (1.0;2.0)].

**Table 3:** Overall impression of each dosage form.

Dosage form	N (%)	Median (Q1;Q3)
Tablets	Very good (1): 8 (12.3%) Good (2): 13 (20.0%) Fair (3): 21 (32.3%) Poor (4): 15 (23.1%) Very poor (5): 8 (12.3%)	3.0 (2.0;4.0)
Eye-drops	Very good (1): 27 (42.2%) Good (2): 28 (43.8%) Fair (3): 5 (7.8%) Poor (4): 3 (4.7%) Very poor (5): 1 (1.6%)	2.0 (1.0;2.0)
Oral drops	Very good (1): 23 (35.4%) Good (2): 15 (23.1%) Fair (3): 16 (24.6%) Poor (4): 5 (7.7%) Very poor (5): 6 (9.2%)	2.0 (1.0;3.0)
Pen	Very good (1): 11 (18.0%) Good (2): 10 (16.4%) Fair (3): 13 (21.3%) Poor (4): 21 (34.4%) Very poor (5): 6 (9.8%)	3.0 (2.0;4.0)
Patches	Very good (1): 8 (12.7%) Good (2): 24 (38.1%) Fair (3): 10 (15.9%) Poor (4): 15 (23.8%) Very poor (5): 6 (9.5%)	2.0 (2.0;4.0)

Data are shown as number (%), median (Q1;Q3).

Table 4 shows the correlation between the sum score and the overall impression rating of each dosage form. We found very high correlations across the board, ranging from 0.8 to 0.9.

**Table 4:** Correlation between sum scores and overall impression of each dosage form.

	Tablets	Eye-drops	Oral drops	Pen	Patches
Kendall’s-Tau correlation coefficient (*p*-value)	0.754 (*p* < 0.001)	0.825 (*p* < 0.001)	0.904 (*p* < 0.001)	0.892 (*p* < 0.001)	0.896 (*p* < 0.001)

## Discussion

Analyzing medication management performance in older multimorbid patients who reported autonomous medication management for five prototypical dosage forms via video-recorded standardized tasks that were evaluated by two independent raters using a standardized and previously validated rating scheme comprising different steps of medication administration and an overall impression, we observed that 84% had handling problems in at least one step of administration for at least one dosage form.

### Comparison with previous evidence

Previous studies assessing medication management can be classified according to their use of performance-based instruments versus self-reported instruments and the use of an individual’s own medication versus a standardized medication regimen (9). Schenk et al. investigated patients’ medication management in a qualitative pilot study comprising video-based medication management performance and an interview in 20 patients (≥ 65 years old, ≥ 5 drugs, independently living). For evaluating medication management performance, patients had to prepare their personal daily medication, while the interview covered six pertinent aspects: medication history, behavior toward the medication, experience with medication management, handling, adherence, and perceived issues or preferences/wishes regarding their therapy. The video recordings of medication management performance and the interview were evaluated by up to 3 raters in a qualitative way, showing that patients’ medication management skills differed from their self-reported skills and patients overestimated their medication management skills (8).

Mortelmans et al. investigated medication management in 400 geriatric patients with polypharmacy (mean age 82 years, 53% women) after hospital discharge by the Medication Management Instrument for Deficiencies in the Elderly (MedMaIDE), covering the three domains medication knowledge, functional ability to take medication, and obtaining medication. The MedMaIDE is a performance-based instrument evaluating the handling of an individual’s own medication. 70% of patients did self-manage medication post-discharge, and 86% had at least one deficiency related to knowledge about the medication. As to the functional abilities, 11% of the patients were unable to open the medication container, 4% were unable to bring the medication up to their mouth, 3% were unable to count the number of pills, and 3% were unable to swallow medication (17). Regarding the ability to open a medication container, the ABLYMED study obtained comparable results: 6% of the patients had handling problems regarding the removal of tablets from the blister pack and 10% had handling problems regarding the removal of tablets from the tablet tube.

In a review, Badawoud et al. found a total of 26 instruments measuring medication self-management capacity. Of those, 14 instruments were performance-based instruments using a standardized medication regimen. Four of these instruments were used for special patient groups (after stroke, with schizophrenia, with HIV). None of the remaining 14 instruments evaluated dosage forms beside tablets and can therefore be meaningfully compared with the ABLYMED study (9), except for the instrument “Patient’s Barriers to Compliance,” which evaluated the ability to open a liquid container (18). The instrument was developed to assist the pharmacist in identifying medication management problems based on a sample of 14 individuals with an average age of 75.5 years. 14% could not open child-resistant containers and 29% could not open child-resistant bottles (18). In the larger ABLYMED study sample, 11% of the patients could not open a child-resistant dropper bottle.

Beckmann et al. assessed hand function (opening bottle), vision (reading label), and medication competence (comprehension and calculation) in a nationally representative sample of persons >77 years in Sweden (*n* = 492). 66.3% of the study participants had at least one limitation in medication management skills, meaning they did not pass at least one of the described tasks (6). The ABLYMED study involved a selected patient sample (≥70 years of age, regularly taking ≥5 different drugs autonomously) and observed handling problems in a similar size.

Previous studies assessing handling problems of other dosage forms that can be compared with our study are limited (9). The instrument “Show Back” is a performance-based instrument using patients’ own medication. It assesses the administration of injectable and inhaled medications as well as tablets. Unfortunately, there were only few patients using pens or inhalers to assess the administration (19). Of note, problems in inhaler handling and their impact on disease exacerbation are well investigated (20).

### Limitations

Despite our innovative study design there are limitations. We included 100 patients in the ABLYMED study, *n* = 67 performed video-recorded medication management and *n* = 57 completed all dosage forms. It can be assumed that patients with medication management problems are more likely to avoid the video recordings resulting in biased results.

Besides, we measured medication management performance of older inpatients in the hospital. A comparison with a control group could be useful to interpret our results. Perhaps medication management skills of inpatients are poor due to the hospital setting. To examine this possible bias, we measure medication management performance of older patients in a general practitioner setting in a follow-up study.

To combine the rating results of two raters into one value for further analyses, we calculated the median value rounded up to the next whole number. This implies that in case of no major discrepancy, that is not more than one category difference between the two raters, the most severe rating is taken. As higher scores indicate greater difficulties in medication management, performance is possibly underrated. However, the interrater agreement between the two raters was satisfactory with an intraclass correlation coefficient (ICC) between 0.67 and 0.99.

Furthermore, our medication management tasks did not cover all steps of medication administration. Due to the protection of data privacy, we did not film the patients’ faces. Thus, for eye-drops, patients were asked to open the one-dose ophtiole dispenser but we could not evaluate the application to the eye.

Additionally, patients performed medication management of all dosage forms independent of their own medication. We did not take into account that some patients had more practice in the dosage forms included in their own medication. In the sample of the ABLYMED study, all patients regularly took tablets, 24% used eye-drops, 22% pens, 7% oral drops and 2% patches.

### Clinical implications

Our study investigated the prevalence of handling problems in older multimorbid patients reporting autonomous medication management. Due to resilience, overconfidence, unwillingness to acknowledge the own limitations, self-confirmation of independence and lack of knowledge on medication, discrepancies between subjective and objective patient performance result (8). Pharmacists and general practitioner must pay attention to these discrepancies and should increase patients’ awareness, because the correct handling of medication is essential for the success of medical treatments. Otherwise, optimal treatment outcomes cannot be ensured (2, 20) and adverse effects may occur. In addition to a poor clinical outcome, incorrect medication management can lead to non-adherence with possible consequences like additional drug therapy or hospitalization (21). Thus, medication management problems must be considered when prescribing medication. The aim of the ABLYMED study is the development of a novel tool to assess the ability to self-administer medication, which can be used by general practitioner also to increase patients’ awareness on medication management problems. Practical consequences could be adaptation of the medication regimen, trainings, and explanations about medication management and finally prescription of nursing support in case of insufficient patient abilities to self-administer medication.

## Conclusion

Evaluating medication management performance in a typical older, non-demented patient population with polypharmacy and autonomous medication management via video-recorded standardized tasks, we were able to reveal important handling problems with medication in five common dosage forms. The evaluation of the videos by two raters with a standardized and previously validated rating scheme provides an objective judgment of the patient’s abilities. People older than 80 years have three diagnoses on average. The medication regimens to treat these diseases are usually complex, including several drugs, different dosage forms and dosage frequencies. Additionally, new drugs may be prescribed at any time, further increasing the management load. Consequently, all common dosage forms should be evaluated with a comprehensive measurement of medication management skills. Due to the gap between patients’ self-reported abilities in medication management and their actual observable skills, a performance-based measure of medication management is strongly recommended for a valid assessment. Therefore, health care providers should evaluate the medication self-management skills of older patients to reduce unintentional non-adherence and associated harms. In a next step, factors that influence the ability to self-administer medication will be analyzed in the ABLYMED study. These factors will then be combined into a short assessment tool that is suitable for everyday use. The assessment tool is designed to help healthcare professionals identify patients who are at risk of suffering from substantial handling problems. These patients could then be offered individual help such as patient-individual trainings or a modification of the prescribed drug regimen.

## Data Availability

The datasets presented in this article are not readily available due to data security. Requests to access the datasets should be directed to JG, janine.gronewold@uk-essen.de.
