# Supplementary material for: Assessing self-administration of medication: video-based evaluation of patient performance in the ABLYMED study
Source: Front Med (Lausanne). 2024 Nov 19;11:1444567. doi: 10.3389/fmed.2024.1444567 (PMC11613422; doi:10.3389/fmed.2024.1444567)
Supplement: Supplementary file 1 [file Data_Sheet_1.PDF]

Supplementary Material

Table S1. Interrater agreement for ratings of each step of medication administration for each dosage form

**Dosage form**	**Differences between rating results from rater JG and rater TD**	**N (%)**	**Weighted Cohen´s kappa**
**Tablets**			
White tablet removal from the blister pack	rater JG 3 lower than rater TD	0 (0.0%)	0.36
	rater JG 2 lower than rater TD	1 (1.5%)	
	rater JG 1 lower than rater TD	19 (29.2%)	
	rater JG and TD total agreement	30 (46.2%)	
	rater JG 1 higher than rater TD	11 (16.9%)	
	rater JG 2 higher than rater TD	3 (4.6%)	
	rater JG 3 higher than rater TD	1 (1.5%)	
Blue tablet removal from the tablet tube	rater JG 3 lower than rater TD	2 (3.1%)	0.40
	rater JG 2 lower than rater TD	1 (1.5%)	
	rater JG 1 lower than rater TD	5 (7.7%)	
	rater JG and TD total agreement	34 (52.3%)	
	rater JG 1 higher than rater TD	16 (24.6%)	
	rater JG 2 higher than rater TD	3 (4.6%)	
	rater JG 3 higher than rater TD	4 (6.2%)	
Cutting the blue tablet	rater JG 3 lower than rater TD	1 (1.5%)	0.67
	rater JG 2 lower than rater TD	4 (6.2%)	
	rater JG 1 lower than rater TD	6 (9.2%)	

	rater JG and TD total agreement	44 (67.7%)	
	rater JG 1 higher than rater TD	7 (10.8%)	
	rater JG 2 higher than rater TD	3 (4.6%)	
	rater JG 3 higher than rater TD	0 (0.0%)	
Correctly filling the pill organizer	rater JG 1 lower than rater TD	2 (3.1%)	0.81
	rater JG and TD total agreement	60 (92.3%)	
	rater JG 1 higher than rater TD	3 (4.6%)	
**Eye-drops**			
Open the one-dose ophtiole dispenser	rater JG 3 lower than rater TD	0 (0.0%)	0.44
	rater JG 2 lower than rater TD	1 (1.6%)	
	rater JG 1 lower than rater TD	15 (23.4%)	
	rater JG and TD total agreement	39 (60.9%)	
	rater JG 1 higher than rater TD	8 (12.5%)	
	rater JG 2 higher than rater TD	1 (1.6%)	
	rater JG 3 higher than rater TD	0 (0.0%)	
**Oral drops**			
Open the child-resistant dropper bottle	rater JG 3 lower than rater TD	0 (0.0%)	0.76
	rater JG 2 lower than rater TD	0 (0.0%)	
	rater JG 1 lower than rater TD	2 (3.1%)	
	rater JG and TD total agreement	49 (75.4%)	
	rater JG 1 higher than rater TD	11 (16.9%)	
	rater JG 2 higher than rater TD	3 (4.6%)	
	rater JG 3 higher than rater TD	0 (0.0%)	

Aiming at the teaspoon	rater JG 3 lower than rater TD<br>rater JG 2 lower than rater TD<br>rater JG 1 lower than rater TD<br>rater JG and TD total agreement<br>rater JG 1 higher than rater TD<br>rater JG 2 higher than rater TD<br>rater JG 3 higher than rater TD<br>rater JG 4 higher than rater TD	0 (0.0%)<br>0 (0.0%)<br>0 (0.0%)<br>47 (72.3%)<br>7 (10.8%)<br>3 (4.6%)<br>5 (7.7%)<br>3 (4.6%)	0.40
Correct number of drops (n=10) on the teaspoon	rater JG 1 lower than rater TD<br>rater JG and TD total agreement<br>rater JG 1 higher than rater TD	0 (0.0%)<br>54 (83.1%)<br>11 (16.9%)	0.60
**Pen**			
Remove the transparent cap of the pen	rater JG 3 lower than rater TD<br>rater JG 2 lower than rater TD<br>rater JG 1 lower than rater TD<br>rater JG and TD total agreement<br>rater JG 1 higher than rater TD<br>rater JG 2 higher than rater TD<br>rater JG 3 higher than rater TD	0 (0.0%)<br>0 (0.0%)<br>3 (4.9%)<br>58 (95.1%)<br>0 (0.0%)<br>0 (0.0%)<br>0 (0.0%)	0.85
Remove the green cap of the needle	rater JG 3 lower than rater TD<br>rater JG 2 lower than rater TD<br>rater JG 1 lower than rater TD<br>rater JG and TD total agreement<br>rater JG 1 higher than rater TD	1 (1.6%)<br>2 (3.3%)<br>2 (3.3%)<br>50 (82.0%)<br>4 (6.6%)	0.76

	rater JG 2 higher than rater TD<br>rater JG 3 higher than rater TD	2 (3.3%)<br>0 (0.0%)	
Dialing in the right dose (12 units)	rater JG 3 lower than rater TD<br>rater JG 2 lower than rater TD<br>rater JG 1 lower than rater TD<br>rater JG and TD total agreement<br>rater JG 1 higher than rater TD<br>rater JG 2 higher than rater TD<br>rater JG 3 higher than rater TD	0 (0.0%)<br>3 (4.9%)<br>3 (4.9%)<br>43 (70.5%)<br>7 (11.5%)<br>4 (6.6%)<br>1 (1.6%)	0.69
Injection into a ball	rater JG 4 lower than rater TD<br>rater JG 3 lower than rater TD<br>rater JG 2 lower than rater TD<br>rater JG 1 lower than rater TD<br>rater JG and TD total agreement<br>rater JG 1 higher than rater TD<br>rater JG 2 higher than rater TD<br>rater JG 3 higher than rater TD	1 (1.6%)<br>1 (1.6%)<br>1 (1.6%)<br>5 (8.2%)<br>39 (63.9%)<br>10 (16.4%)<br>2 (3.3%)<br>2 (3.3%)	0.55
**Patches**			
Unpacking the patch	rater JG 3 lower than rater TD<br>rater JG 2 lower than rater TD<br>rater JG 1 lower than rater TD<br>rater JG and TD total agreement<br>rater JG 1 higher than rater TD<br>rater JG 2 higher than rater TD	0 (0.0%)<br>3 (4.8%)<br>7 (11.1%)<br>46 (73.0%)<br>3 (4.8%)<br>4 (6.3%)	0.54

	rater JG 3 higher than rater TD	0 (0.0%)	
Peeling off the protective liner	rater JG 3 lower than rater TD	0 (0.0%)	0.64
	rater JG 2 lower than rater TD	1 (1.6%)	
	rater JG 1 lower than rater TD	7 (11.1%)	
	rater JG and TD total agreement	38 (60.3%)	
	rater JG 1 higher than rater TD	10 (15.9%)	
	rater JG 2 higher than rater TD	5 (7.9%)	
	rater JG 3 higher than rater TD	2 (3.2%)	
Applying the patch onto the skin	rater JG 4 lower than rater TD	1 (1.6%)	0.64
	rater JG 3 lower than rater TD	1 (1.6%)	
	rater JG 2 lower than rater TD	1 (1.6%)	
	rater JG 1 lower than rater TD	10 (15.9%)	
	rater JG and TD total agreement	45 (71.4%)	
	rater JG 1 higher than rater TD	5 (7.9%)	
	rater JG 2 higher than rater TD	0 (0.0%)	
	rater JG 3 higher than rater TD	0 (0.0%)	

Data are shown as number (%) and weighted Cohen´s kappa.

Table S2. Interrater agreement for ratings of the overall impression of each dosage form

**Dosage form**	**Differences between rating results from rater JG and rater TD**	**N (%)**	**Weighted Cohen´s kappa**
Tablets	rater JG 3 lower than rater TD	0 (0.0%)	0.53
	rater JG 2 lower than rater TD	3 (4.6%)	
	rater JG 1 lower than rater TD	1 (1.5%)	
	rater JG and TD total agreement	29 (44.6%)	
	rater JG 1 higher than rater TD	28 (43.1)	
	rater JG 2 higher than rater TD	3 (4.6%)	
	rater JG 3 higher than rater TD	1 (1.5%)	
Eye-drops	rater JG 3 lower than rater TD	0 (0.0%)	0.52
	rater JG 2 lower than rater TD	1 (1.6%)	
	rater JG 1 lower than rater TD	8 (12.5%)	
	rater JG and TD total agreement	40 (61.5%)	
	rater JG 1 higher than rater TD	14 (21.5%)	
	rater JG 2 higher than rater TD	1 (1.6%)	
	rater JG 3 higher than rater TD	0 (0.0%)	
Oral drops	rater JG 3 lower than rater TD	0 (0.0%)	0.39
	rater JG 2 lower than rater TD	0 (0.0%)	
	rater JG 1 lower than rater TD	0 (0.0%)	
	rater JG and TD total agreement	29 (44.6%)	
	rater JG 1 higher than rater TD	17 (26.2%)	
	rater JG 2 higher than rater TD	12 (18.5%)	
	rater JG 3 higher than rater TD	5 (7.7%)	

	rater JG 4 higher than rater TD	2 (3.1%)	
Pen	rater JG 3 lower than rater TD	0 (0.0%)	0.41
	rater JG 2 lower than rater TD	0 (0.0%)	
	rater JG 1 lower than rater TD	3 (4.9%)	
	rater JG and TD total agreement	20 (32.8%)	
	rater JG 1 higher than rater TD	23 (37.7%)	
	rater JG 2 higher than rater TD	12 (19.7%)	
	rater JG 3 higher than rater TD	3 (4.9%)	
Patches	rater JG 3 lower than rater TD	0 (0.0%)	0.62
	rater JG 2 lower than rater TD	0 (0.0%)	
	rater JG 1 lower than rater TD	3 (4.8%)	
	rater JG and TD total agreement	37 (58.7%)	
	rater JG 1 higher than rater TD	15 (23.8%)	
	rater JG 2 higher than rater TD	6 (9.5%)	
	rater JG 3 higher than rater TD	2 (3.2%)	

Data are shown as number (%) and weighted Cohen´s kappa.
